# Protective Effects of Dexmedetomidine and Amifostine Against Radiotherapy-Induced Kidney Injury

**DOI:** 10.3390/life15060897

**Published:** 2025-05-31

**Authors:** Sule Batcik, Levent Tumkaya, Eyup Dil, Leyla Kazancioglu, Elif Gaygusuz, Zihni Acar Yazici, Zulkar Ozden, Kagan Kilinc, Tolga Mercantepe

**Affiliations:** 1 Department of Anesthesiology and Reanimation, Faculty of Medicine, Recep Tayyip Erdogan University, 53100 Rize, Turkey; leyla.kazancioglu@erdogan.edu.tr; 2 Department of Histology and Embryology, Faculty of Medicine, On Dokuz Mayis University, 55010 Samsun, Turkey; tumkaya55@hotmail.com; 3 Department of Urology, Faculty of Medicine, Recep Tayyip Erdogan University, 53100 Rize, Turkey; eyup.dil@erdogan.edu.tr; 4 Department of Biochemistry, Faculty of Medicine, Recep Tayyip Erdogan University, 53100 Rize, Turkey; elif_gaygusuz24@erdogan.edu.tr; 5 Department of Medical Microbiology, Faculty of Medicine, Recep Tayyip Erdogan University, 53100 Rize, Turkey; 6 Department of Histology and Embryology, Faculty of Medicine, Recep Tayyip Erdogan University, 53100 Rize, Turkey; zulkar.ozden@saglik.gov.tr (Z.O.); tolgamercantepe@yahoo.com (T.M.); 7 Department of Genetic and Bioengineering, Faculty of Engineering and Natural Sciences, Gumushane University, 29000 Gumushane, Turkey; kagankilinc@yahoo.com

**Keywords:** amifostine, alpha 2 adrenergic agonists, kidney, x-irradiation

## Abstract

*Backgrounds*: Approximately 18 million individuals were diagnosed with cancer in 2018. The rate is predicted to exceed 22 million by 2030. Radiotherapy is an essential part of cancer therapy, with well documented local and systemic side effects, including oxidative stress and apoptosis. Kidney tissues are also exposed to the deleterious effects of radiotherapy, resulting in acute or chronic kidney function impairment. This study compared the effects of the potent selective α2-adrenoreceptor agonist dexmedetomidine and amifostine on oxidative stress and apoptosis in kidney damage induced by x-irradiation in rats. *Methods*: Forty Sprague Dawley rats were assigned into five groups: control, x-irradiation, x-irradiation + amifostine, x-irradiation + dexmedetomidine 100 µg/kg, and X-ray irradiation + dexmedetomidine 200 µg/kg. *Results*: Necrotic tubules and degenerative Bowman’s capsules were present in the x-irradiation group. An increase was determined in malondialdehyde (MDA), Cleaved Caspase-3, and 8-OHdG levels compared to the control group (*p* ≤ 0.05). In contrast, there was a decrease in necrotic tubules, degenerative Bowman’s capsules, and the levels of MDA, Cleaved Caspase-3, and 8-OHdG in the amifostine and dexmedetomidine 100 µg/kg and 200 µg/kg treatment groups (*p* ≤ 0.05). *Conclusions*: Alpha 2 adrenergic receptor agonists exhibit protective effects against kidney injury induced in association with x-irradiation by reducing oxidative stress and apoptosis.

## 1. Introduction

Cancer is among the most common causes of mortality globally. In 2018, around 18 million people received a cancer diagnosis, and over 9 million deaths were linked to the disease [1]. By 2030, it is predicted that the number of patients with cancer will exceed 22 million [2,3]. Radiotherapy is an essential part of cancer treatment. Radiotherapy is applied in approximately 50% of cancer cases and exhibits healing effects on approximately 30–40% of patients [4]. X-irradiation, which is generally applied during radiotherapy, penetrates powerfully into the body and is dispersed in subcutaneous tissue. This means that healthy tissues in the area out of focus are also exposed to radiation during the irradiation of cancerous tissue. Although normal cells are generally capable of tolerating a large part of the radiation, irradiation is known to damage healthy tissues [5]. Kidney tissues are also exposed to the deleterious effects of irradiation during radiotherapy, and acute or chronic kidney function impairment occurs as a result. Radiation-related kidney function impairment is inevitable in patients undergoing whole-body irradiation, particularly before radionuclide therapy and bone marrow transplantation [6,7].

The mechanisms concerning the molecular and cellular processes involved in radiation-related nephrotoxicity are not yet fully understood. However, experimental studies indicate that DNA damage, the main target of radiotherapy, results from oxidative stress induced by reactive oxygen species (ROS) [8,9,10]. The elevation of reactive oxygen species (ROS), which can damage macromolecules including DNA, lipids, and proteins due to ionizing radiation, results in the dysfunction of antioxidant enzyme systems, hence initiating oxidative stress [11,12]. Studies have indicated that increased ROS due to irradiation impairs antioxidant mechanisms, resulting in raised malondialdehyde (MDA) levels and significant reductions of glutathione (GSH) levels [13]. Furthermore, the elevation of ROS can cause DNA oxidation, leading to increased 8-hydroxy-2′-deoxyguanosine (8-OHdG) levels in renal tissue [14]. Studies have also shown that injury occurring in DNA can lead to apoptosis in renal tissue by elevating caspase-3 activation [9]. Impairments in cellular mechanisms associated with irradiation are easily identified in acute kidney injury. Abnormal histological changes at the renal glomerular and tubular structures result largely from cell death in the forms of apoptosis and necrosis [15,16].

Amifostine is a prodrug that is metabolized into an active sulfhydryl compound by alkaline phosphatase, which is capable of selectively protecting against free radical-induced radiation by scavenging free radicals [17,18]. Amifostine has been reported to exhibit ionizing-radiation-induced protective effects including kidneys, bone marrow, and lungs [19,20,21,22,23].

Dexmedetomidine (DEX), a highly selective α2-adrenoreceptor agonist, is a drug that is often used clinically for analgesia and sedation [24]. Studies investigating the antioxidant properties of DEX have shown that it can reduce lipid peroxidation and cause an improvement in antioxidant enzyme activities. Recent studies indicate that DEX exhibits therapeutic effects by diminishing the production of inflammatory cytokines in renal tissues, which elevate ROS, and enhancing apoptosis, with its protective effects also linked to the mitigation of oxidative stress [25,26,27]. However, the impact of DEX on X-ray-induced renal injury is unknown.

This research compared the effects of the potent selective α2-adrenoceptor agonist dexmedetomidine and amifostine on oxidative stress and apoptosis using histochemical, biochemical, and immunohistochemical methods in kidney damage induced by x-irradiation rats.

## 2. Materials and Methods

### 2.1. Animals

Forty male Sprague Dawley rats weighing 350–390 g and aged 4–5 months were used in this study. The rats were obtained from the Experimental Animals Unit of Recep Tayyip Erdoğan University. During the experiment, the rats were housed in a unit where optimal conditions were provided, including a 12 h light–dark cycle, a humidity level of 55–60%, and a temperature of 22 ± 2 °C. The rats had ad libitum access to tap water and pellet chow. This experimental study was approved by Recep Tayyip Erdogan University’s Ethical Council for Animal Research. The study was performed in consideration of factors affecting the quality of life and well-being of experimental animals (Rize, Türkiye, No. 202, dated 1 October 2021).

### 2.2. Experimental Design

Rats were randomly assigned into five groups of 8, using a numbering program loaded onto a computer. The groups were control, x-irradiation, x-irradiation + amifostine (AMF), x-irradiation + Dex100 (Dex 100 µg), and x-irradiation + Dex200 (Dex 200 µg). X-ray irradiation was applied to the rats in the X-irradiation group. X-irradiation + AMF, X-irradiation + Dex 100, and X-irradiation + Dex 200 groups of rats were administered 200 µg/kg amifostine (Ethyol 500 mg/1 flacon, Er-Kim, Istanbul, Türkiye), 100 µg/kg dexmedetomidine (Precedex, USA), and 200 µg/kg dexmedetomidine (Precedex, USA) intraperitoneally 30 min before X-ray exposure, respectively. The control group rats received a single dose of saline solution (1 mL) intraperitoneally.

### 2.3. X-Irradiation Protocols

The X-irradiation procedure was performed it the Oncology Department, Recep Tayyip Erdogan University Medical Faculty. Before irradiation, rats were anesthetized by administering 50 mg/kg of ketamine (Ketalar, Pfizer, Istanbul, Türkiye) and 5 mg/kg of xylazine (Rompun, Bayer, Türkiye) i.p. Rats under anesthesia were placed in a prone position on the unit. Conformal planning was performed before radiation application with CMS Xio (version: 5.0; Elekta, Stockholm, Sweden). External radiotherapy of 8 Gy was applied using Elekta Synergy linear accelerator in a single fraction, using 6 MV energy, at a distance of 100 cm from the front of the skin, such as to affect the rats’ head–neck regions. Rats were given high-dose anesthesia. They were euthanized twenty-four hours after irradiation to better observe radiation-induced acute nephrotoxicity [15].

#### Biochemical Analysis

A mass of 100 mg renal tissues was homogenized in 1 mL of PBS (pH 7.4). The homogenates were centrifuged at 800× *g* for 10 min at 4 °C. The supernatant was retained for GSH and MDA measurements.

### 2.4. Measurement of Antioxidant Enzyme Activities

MDA analysis in kidney tissues was performed as described [28]. A volume of 200 µL of supernatants were added to 50 µL of sodium dodecyl sulfate (SDS, 8.1%), 375 µL of acetic acid (20%, pH 3.5), and 375 µL thiobarbituric acid (TBA, of 0.8%). The homogenized samples were incubated in a hot water bath for 1 h. After incubation, the samples were cooled in icy water for 5 min and centrifuged at 750× *g* for 10 min. Color change was measured spectrophotometrically at 532 nm and the results were expressed as nmol/g tissue.

Kidney GSH was measured by using the Ellman method [29]. 100 µL of 3M Na_2_HPO_4_ and 25 µL of 5,5′-dithiobis (2-nitrobenzoic acid) (DTNB) (4 mg DTNB prepared in 10 mL of 1% sodium citrate solution) were added to 25 µL of the supernatant. Then, the solution was gently shaken, and the yellow color formed after the reaction was measured at a wavelength of 412 nm using a spectrophotometer. The results were calculated using a reduced GSH standard curve of 1000 µM–62.5 µM and expressed as mmol/g tissue.

### 2.5. Histopathological Analysis

The kidney tissues were promptly excised and fixed in 10% formalin for 24 h for histopathological and immunohistochemical analyses. Tissue tracking procedures following fixation were conducted using the Citadel 2000 Shandon (Thermo Scientific, Cheshire, UK) autotechnicon device. The tissues underwent dehydration by passing through a series of increasing alcohol concentrations, followed by treatment with xylene for the clearing process. Subsequently, it was embedded in paraffin and blocked. Sections 3–4 µm in thickness were taken from paraffin blocks and stained using Harris hematoxylin and eosin G (H&E) for structural examination. The kidney slides were analyzed using a light microscope and captured in photographs (Olympus DP71 BX51, Olympus Corporation, Tokyo, Japan).

### 2.6. Immunohistochemical (IHC) Analysis

Kidney tissues were fixed in 10% formalin and embedded in paraffin. 2–3 µm sections were taken and placed on poly-l-lysine-coated slides. Following the deparaffinization process, staining of the sections was performed with 8-OHdG (sc-66036, diluted 1:200, Santa Cruz, Europe) and caspase-3 (diluted 1:200, caspase-3/p17, SC373730, Santa Cruz Biotechnology, Inc., USA) antibodies using a Bond-Max model (Leica Biosystems) automatic immunohistochemical staining device. The slides were deparaffinized with Bond Dewax solution for that purpose. Peroxidase blocking was then performed on the dehydrated kidney tissues. Antigen retrieval was carried out with 20 min heating in ER2 (Leica Biosystems) solution. Incubation with 8-OHdG and caspase-3 antibodies was then performed for 60 min. DAB in the Bond Polymer Refine Detection kit (Leica Biosystems) was dropped onto the tissues treated with secondary antibodies, and the preparations were stained with hematoxylin (Bond Polymer Refine Detection, Leica) for 10 min. After staining with hematoxylin, the slides were mounted using Entellan and examined and photographed. In terms of immunohistochemistry, a semi-quantitative analysis of immunoreactivity in kidney tissues stained with 8-OHdG and caspase-3 antibodies was performed with Mercantepe et al.’s scoring method [30]. Accordingly, immunopositivity scoring was conducted using the blinding method by two independent histopathologists—1 (mild; ≤5%), 2 (moderate; ≥10%), 3 (severe; ≥25%), and 4 (extremely severe; ≥50%).

### 2.7. Semi-Quantitative Analysis

A Kidney Histopathologic Damage Score (KHDS) was established, based on the findings of previous studies involving ionizing radiation-induced kidney injury and Jeong Sun et al.’s Histopathological Kidney Injury Score for tubular necrosis for the histological analysis of H&E-stained renal tissue sections (Table 1) [31]. Semi-quantitative analyses involving 20 distinct areas in preparations from each rat were carried out by two histopathologists blinded to the study groups.

### 2.8. Statistical Analysis

Data were analyzed using the SPSS 18.00 software (IBM Corp. Chicago, IL, USA). Data yielded by semi-quantitative analyses were expressed as median and 25th and 75th interquartile values. Differences between groups were evaluated using the Kruskal–Wallis and Tamhane T2 tests. Data yielded by quantitative analyses were calculated as arithmetic mean ± standard deviation. Differences between groups were examined using one-way ANOVA and the Tukey test. *p* values < 0.05 were regarded as statistically significant.

## 3. Results

### 3.1. Biochemical Results

Malondialdehyde (MDA) levels in kidney tissue were elevated in the X-irradiation group in comparison to the control group (*p* < 0.05, Table 2). Conversely, levels of MDA were diminished in the amifostine (AMF) group in comparison to the x-irradiation group (*p* < 0.05, Table 2). Similarly, the MDA levels decreased in the Dex 100 mg and Dex 200 mg treatment groups compared to the x-irradiation groups (*p* < 0.05, Table 2).

The GSH levels were lower in the x-irradiation group than those of the control group (Table 2, *p* < 0.05). In contrast, GSH levels increased in the AMF group compared to the x-irradiation group (Table 2, *p* < 0.05). Similarly, tissue GSH levels increased in the Dex 100 mg and Dex 200 mg treatment groups compared to the x-irradiation group (*p* < 0.05, Table 2).

### 3.2. Histopathological Analysis

**Figure 1:**
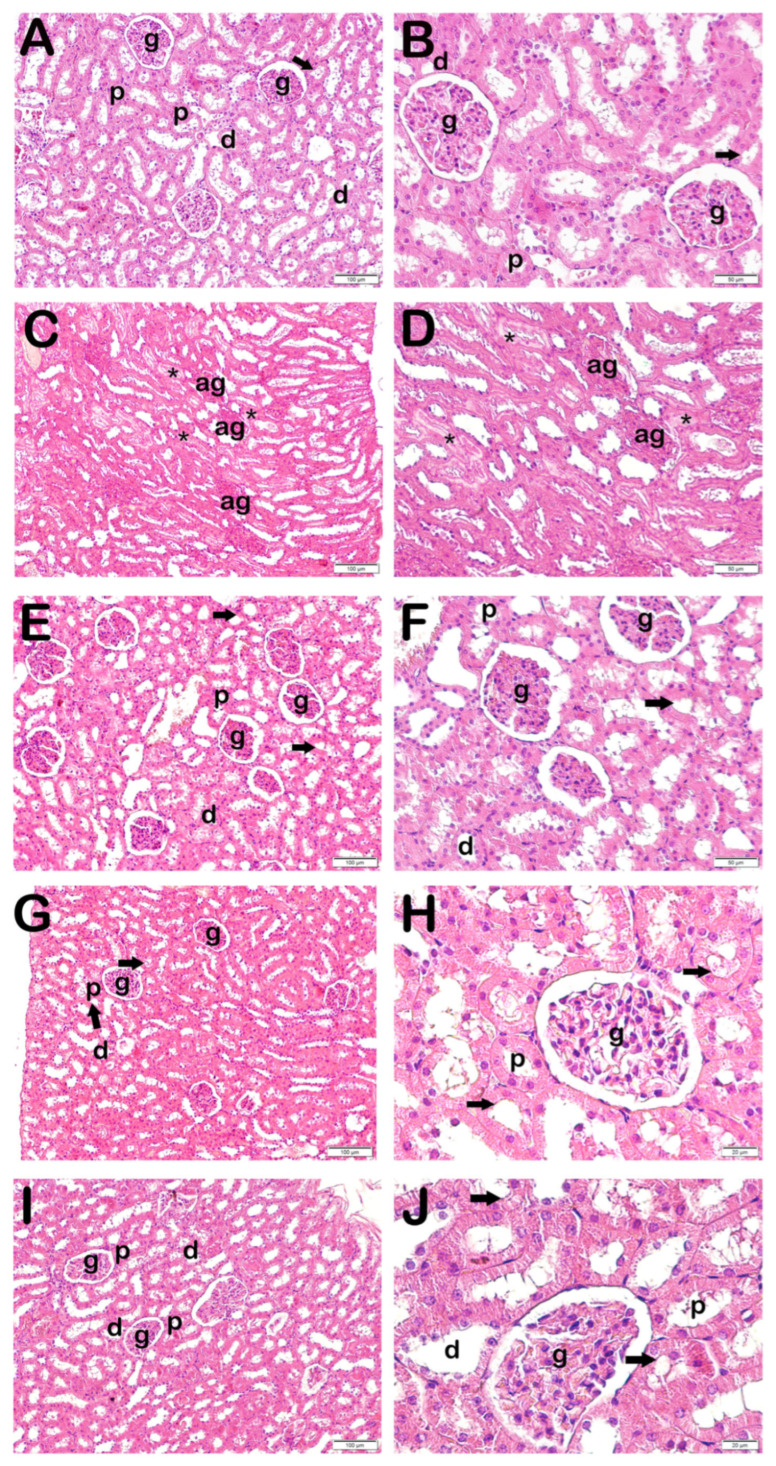
Representative light microscopic image of kidney tissue stained with H&E. Proximal tubule (p), distal tubule (d), glomerulus (g), brush border (arrow), atypical glomeruli (ag), necrotic tubules accompanying debris accumulation in lumens (star). (**A**,**B**) **Controls**: Normal renal tubule and glomerulus (g) structures can be seen. Brush border in proximal tubule has a typical structure (arrow) (KHDS: 0.5 (0–1)). (**C**,**D**) **X-ray irradiation group**: Widespread atypical glomeruli (ag) and necrotic tubules accompanying debris accumulation in lumens (asterisk) (KHDS: 6.5 (6–7)). (**E**,**F**) **AMF treatment group**: Decreases can be seen in degenerative glomeruli and necrotic tubules (KHDS: 1.0 (1–3)). (**G**,**H**) **Dex 100 µg treatment group**: Decreases can be seen in necrotic tubules accompanying debris accumulations in lumens and atypical glomerular structures (KHDS: 1.0 (1–2)). (**I**,**J**) **Dex 200 µg treatment group**: Tubules and glomerular structures are typical in appearance (KHDS: 1.0 (1–1)).

In the light microscopic examinations of H&E-stained kidney tissues, the Bowman’s capsules exhibited a normal glomerular structure and the typical kidney tubules in the control group. Furthermore, proximal tubules exhibiting a brush border morphology were noted (Figure 1 and Table 3). Conversely, a degenerative Bowman’s capsule associated with atypical glomeruli was seen in the x-irradiation group.

Necrotic tubules accompanying widespread luminal debris accumulations were also observed. The loss of the brush border in proximal tubules, in particular, was also evident (Figure 1, Table 3). Decreased necrotic tubules and degenerative Bowman’s capsules were observed in the AMF group (Figure 1, Table 3). Decreased necrotic tubules and Bowman’s capsule structures were also seen in the Dex 100 mg and 200 groups (Figure 1, Table 3). Specifically, the proximal tubule brush border structures were found to be normal (Figure 1, Table 3).

### 3.3. Semi-Quantitative Results

When KHDS scores were calculated in the light of necrotic tubule findings accompanying debris accumulation in renal tubules and loss of the brush border in proximal tubules in H&E-stained kidney tissue sections, together with findings of degenerative Bowman’s capsules accompanying atypical glomeruli, the KHDS score of 0.50 (0–1) in the control group rose to 6.50 (6–7) in the x-irradiation group (Figure 1A,B, Table 4, *p* = 0.000). In contrast, the KHDS score of 6.50 (6–7) in the x-irradiation group decreased to 1.00 (1–3) in the AMF group (Figure 1B,C, Table 4, *p* = 0.000). Similarly, the KHDS score of 6.50 (6–7) in the x-irradiation group decreased to 1.00 (1–2) in the Dex 100 mg group and to 1.00 (1–1) in the Dex 200 mg group (Figure 1B,D–E, Table 4, *p* = 0.000 and *p* = 0.000, respectively).

### 3.4. Immunohistochemical Results

**Figure 2:**
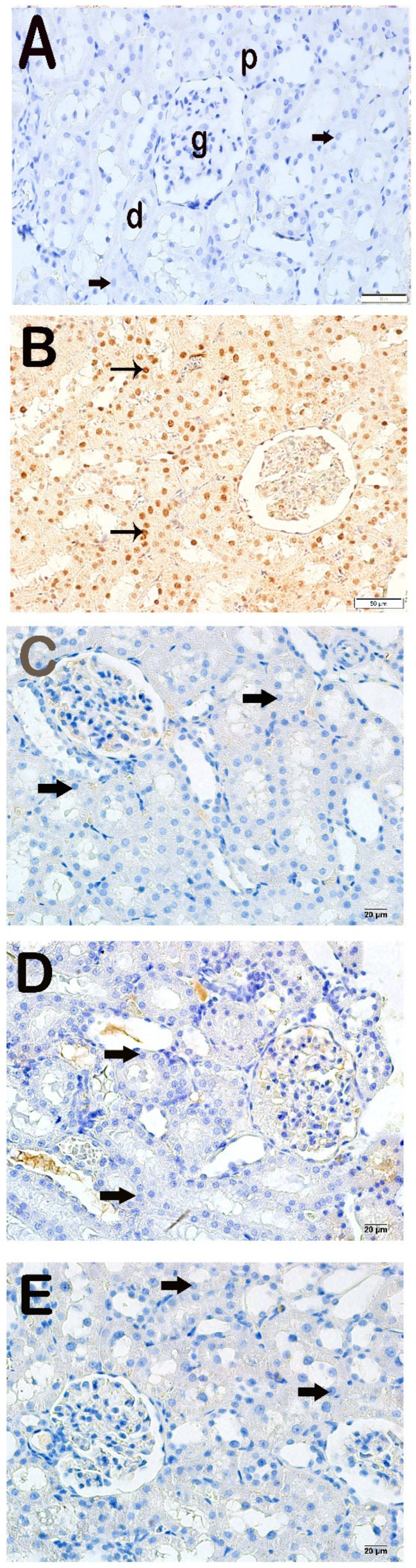
Representative light microscopic appearance of kidney sections incubated with cleaved Caspase-3. Proximal tubule (p), distal tubule (d), glomerulus (g). (**A**) **Control group**: Normal renal tubule epithelial cells (arrow)**.** (Cleaved Caspase-3 positivity score: 0.0 (0–1)). (**B**) **X-irradiation group**: Intense cleaved Caspase-3 positivity in apoptotic renal tubule epithelial cells of (tailed arrow) (Cleaved Caspase-3 positivity score: 2.50 (2–3)). (**C**) **X-irradiation + AMF treatment group**: The number of cells showing Cleaved Caspase-3 positivity was decreased in renal tubule apoptotic epithelial cells (tailed arrow) (cleaved Caspase-3 positivity score: 0.50 (0–1)). (**D**) **X-irradiation + Dex 100 µg treatment group**: Cleaved Caspase-3-positive cells have decreased in renal tubule epithelial cells (tailed arrow) (cleaved Caspase-3 positivity score: 0.00 (0–1)). (**E**) **X-irradiation + Dex 200 µg treatment group**: Cleaved Caspase-3-positive cells have decreased in typical renal tubule epithelial (tailed arrow) (cleaved Caspase-3 positivity score: 0.00 (0–1)).

Apoptotic epithelial cells with increased Cleaved Caspase-3 positivity were observed in the x-irradiation group compared to the control group (Figure 2A,B, Table 4, *p* = 0.000). In contrast, decreased Cleaved Caspase-3 positivity was determined in the proximal and distal tubule cells in the AMF group compared to the x-irradiation group (Figure 2B,C, Table 4, *p* = 0.001). Similarly, decreased Cleaved Caspase-3 positivity was determined in renal tubule epithelial cells in the Dex 100 mg and Dex 200 mg groups compared to the x-irradiation groups (Figure 2B–E, Table 4, *p* = 0.001, and *p* = 0.001, respectively).

**Figure 3:**
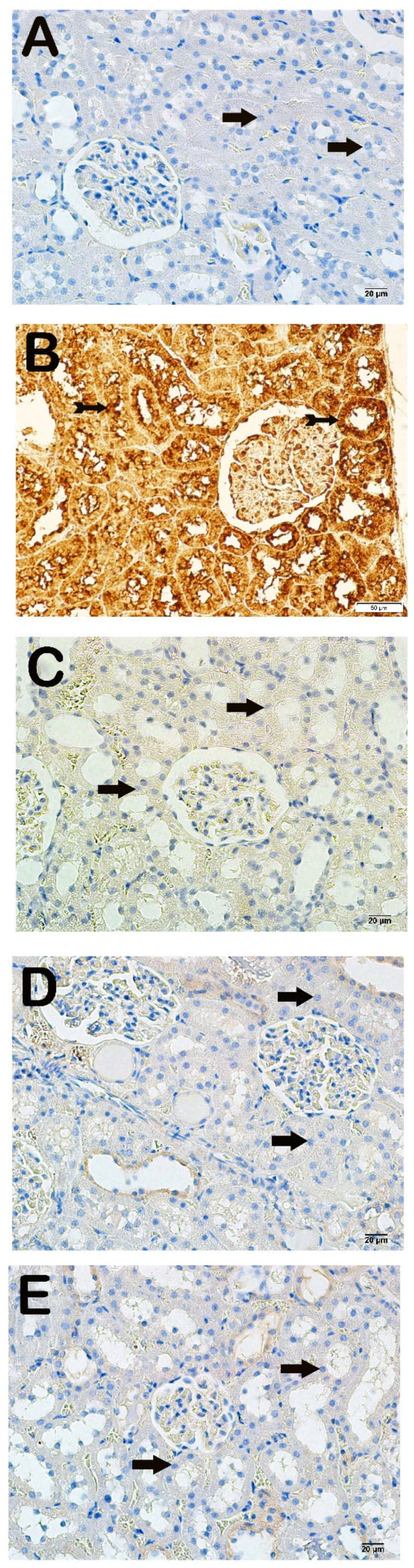
Representative microscopic image of kidney tissue sections incubated with 8-OHdG. (**A**) **Control group**: Normal proximal and distal tubule epithelial cells (arrow) (8-OHdG positivity score: 0.0 (0–1)). (**B**) **X-irradiation group**: Intense 8-OHdG-positivity in proximal and distal tubule epithelial cells (tailed arrow) (8-OHdG positivity score: 3.00 (2–3)). (**C**) **X-irradiation + AMF treatment group**: Decreased 8-OHdG-positivity in renal tubule epithelial cells (tailed arrow) (8-OHdG positivity score: 1.00 (1–2)). (**D**) **X-irradiation + Dex 100 µg treatment group**: Decreased 8-OHdG-positive cells in proximal and distal tubule epithelial cells (tailed arrow) (8-OHdG positivity score: 0.00(0–1)). (**E**) **X-irradiation + Dex 200 µg treatment group**: Decreased 8-OHdG-positive cells in typical renal tubule epithelial cells (tailed arrow) (8-OHdG positivity score: 0.00 (0–1)).

Light microscopic examination of kidney tissues incubated with 8-OHdG primary antibody revealed increased 8-OHdG positivity in the proximal and distal tubule epithelial cells in the x-irradiation group compared to the control group (Figure 3A,B, Table 4, *p* = 0.000). The 8-OHdG positivity in renal tubule cells decreased in the AMF group compared to the x-irradiation group (Figure 3B,C, Table 4, *p* = 0.001). Similarly, 8-OHdG positivity in proximal and distal renal tubules decreased in the Dex 100 mg and Dex 200 mg groups compared to the x-irradiation group (Figure 3B–E, Table 4, *p* = 0.000 and *p* = 0.001, respectively).

## 4. Discussion

Although radiation therapy is an effective method to manage tumor proliferation and prolong survival, it adversely affects healthy tissues in the treatment area. Since kidney tissues are highly radiosensitive, radiotherapy has been reported to cause nephrotoxicity [32,33,34]. Previous studies have investigated the effects of dexmedetomidine and amifostine on cancer tissue [1,2,3,4,5,6,7,8,9,10,11,12,13,14,15,16,17,18,19,20,21,22,23,24]. However, this study is the first to investigate the effects of dexmedetomidine and amifostine on radiotherapy-induced nephrotoxicity. In the present study, DEX caused significant structural and biochemical changes in kidney tissue by preventing oxidative stress and suppressing apoptosis in nephrocytes in rats. DEX had a more protective effect than amifostine on x-irradiation-induced nephrotoxicity.

The mechanism underlying the toxicity of x-irradiation in healthy tissues remains inadequately understood; however, previous studies indicated that this toxicity is associated with oxidative stress [35]. The application of ionizing radiation impairs the balance between oxidant and the antioxidant system by elevating ROS production in tissues. Previous studies indicated that oxidative stress caused by elevated ROS levels can result in kidney injury [36]. MDA, an indirect indicator of the extent of damage produced by free radicals, serves as a significant biomarker for oxidative injury. GSH is a crucial antioxidant in oxidative stress developing in association with x-irradiation [37]. Studies have shown that the application of ionizing radiation increases MDA and decreases GSH activity in various tissues, thereby affecting the oxidant/antioxidant system [9,35,38].

The results of the current study indicate that acute stress compromised the efficacy of the antioxidant mechanism by elevating MDA levels and diminishing GSH enzyme levels.

However, DEX administration resulted in the suppression of oxidative stress with a reduction in MDA activities and an elevation in GSH. Consistent with the present study, Wang et al. indicated that DEX application reduced MDA activity in patients with acute renal conditions [39]. Similarly, Akpinar et al. also demonstrated that DEX possesses antioxidant and oxidative stress-lowering effects by reducing MDA levels and enhancing GSH activities in rat tissues [40].

Studies show that the increase in ROS caused by ionizing radiation results in histopathological changes. Previous studies reported that total body irradiation in rats causes pathological changes in renal tissues, such as degeneration in renal tubules and glomeruli, leukocyte infiltration, and necrosis [30,32,41]. Similarly, in the present study, amifostine reduced the numbers of necrotic tubules and degenerative Bowman’s capsules.

8-OHdG is a recognized biomarker for DNA oxidation caused by ROS generation [42,43,44]. Özyurt et al. observed an increase in 8-OHdG levels in rats exposed to total body 800 cGy irradiation. Similarly, in our study, immunohistochemical analyses showed that x-irradiation led to an increase in 8-OHdG immune reactivity. This condition suggests that DNA is a special target of oxidative damage caused by radiation. In the current study, DEX decreased the levels of 8-OHdG that were elevated due to x-irradiation.

Studies have reported that x-irradiation triggers apoptosis in tissues [9,35]. X-irradiation significantly elevated caspase-3 activity, which is implicated in the terminal phase of apoptosis. Consistent with our research, Mercantepe et al. indicated that a single dose of whole-body 6-Gy irradiation in rats induced apoptosis via elevating the expression of caspase-3 in tubular cells in the kidneys [30]. Previous studies have indicated that DEX reduces apoptosis in the liver, heart, and kidney tissues.

Our study is a pilot study of the effects of dexmedetomidine and amifostine on radiation-induced nephrotoxicity. In addition, our study has some limitations. Our study should be supported by studies that evaluate other antioxidant/oxidant proteins and enzymes and intracellular calcium levels.

In an acute stress-induced model of kidney injury involving Wistar rats, the administration of DEX was observed to inhibit apoptosis through a reduction in the expression of cleaved caspase-3 [45]. However, the impact of DEX on apoptosis in kidney damage induced by x-irradiation remains uncertain. Our study showed that alpha 2 adrenergic receptor agonists have a protective effect against x-irradiation-induced acute kidney injury by reducing oxidative stress and apoptosis.

## Figures and Tables

**Table 1:** Kidney Histopathological Damage Score (KHDS).

Type of Damage	Score	Findings
Deterioration of Brush Border Structure in Proximal Tubules	0	≤5%
	1	6–25%
	2	26–50%
	3	>50%
Loss of tubular epithelial cells connections (debris accumulation in the lumen)	0	≤5%
	1	6–25%
	2	26–50%
	3	>50%
Degenerative glomerulus	0	≤5%
	1	6–25%
	2	26–50%
	3	>50%

**Table 2:** Biochemical analysis results (mean ± standard deviation).

Group	MDA (nmol/mg Tissue)	GSH (nmol/mg Tissue)
Control	0.74 ± 0.26	53.74 ± 2.65
X-ray irradiation	1.86 ± 0.27 **^a^**	38.51 ± 1.94 **^a^**
X-ray irradiation + AMF	0.86 ± 0.26 **^a,b^**	47.55 ± 1.85 **^a,b^**
X-ray irradiation + Dex 100 mg	0.81 ± 0.24 **^a,b^**	47.75 ± 2.4 **^a,b^**
X-ray irradiation + Dex 200 mg	0.82 ± 0.27 **^a,b^**	50.25 ± 1.84 **^a,c^**

^a^ *p* = 0.000 versus control group, ^b^ *p* = 0.000 versus X-irradiation group, ^c^ *p* = 0.022 versus control group, one-way ANOVA/Tukey HSD test.

**Table 3:** Kidney Histopathological Damage Score (KHDS) analysis results (median (25–75%)).

	Brush Border Damage Score	Luminal Debris Accumulation Score	Degenerative Glomerulus Score	KHDS
Control	0.00 (0–0)	0.00 (0–0)	0.00 (0–0)	0.50 (0–1)
X-irradiation	2.50 (2–3) **^a^**	2.00 (2–2) **^d^**	2.00 (2–2) **^d^**	6.50 (6–7) **^a^**
X-ray irradiation + AMF	1.00 (0–1) **^b^**	1.00 (0–1) **^e^**	0.00 (0–1) **^f^**	1.00 (1–3) **^c^**
X-ray irradiation + Dex 100 mg	0.50 (0–1) **^b^**	1.00 (0–1) **^e^**	0.00 (0–1) **^f^**	1.00 (1–2) **^c^**
X-ray irradiation + Dex 200 mg	0.00 (0–1) **^c^**	1.00 (0–1) **^e^**	0.00 (0–0) **^g^**	1.00 (1–1) **^c^**

^a^ *p* = 0.000 versus control group, ^b^ *p* = 0.001 versus X-irradiation group, ^c^ *p* = 0.000 versus X-irradiation group, ^d^ *p* = 0.003 versus control group, ^e^ *p* = 0.027 versus X-irradiation group, ^f^ *p* = 0.006 versus X-irradiation group, ^g^ *p* = 0.003 versus X-irradiation group, Kruskal–Wallis/Tamhane’s T2 test.

**Table 4:** Immunohistochemical (IHC) positive score results (median-(25–75%)).

Group	Cleaved Caspase-3 Positivity	8-OHdG Positivity
Control	0.00 (0–0)	0.00 (0–1)
X-irradiation	2.50 (2–3) **^a^**	3.00 (2–3) **^a^**
X-ray irradiation + AMF	0.50 (0–1) **^b^**	1.00 (1–2) **^b^**
X-ray irradiation + Dex 100 mg	0.00 (0–1) **^b^**	0.00 (0–1) **^c^**
X-ray irradiation + Dex 200 mg	0.00 (0–1) **^b^**	0.00 (0–1) **^c^**

^a^ *p* = 0.000 versus control group, ^b^ *p* = 0.001 versus X-irradiation group, ^c^ *p* = 0.000 versus X-irradiation group, Kruskal–Wallis/Tamhane’s T2 test.

## Data Availability

The corresponding author is able to provide the datasets that were generated and/or analyzed during the present study upon reasonable request.
